# Cooperation Between Systemic and Mucosal Antibodies Induced by Virosomal Vaccines Targeting HIV-1 Env: Protection of Indian Rhesus Macaques Against Low-Dose Intravaginal SHIV Challenges

**DOI:** 10.3389/fimmu.2022.788619

**Published:** 2022-02-22

**Authors:** Samir K. Lakhashe, Mario Amacker, Dinesh Hariraju, Hemant K. Vyas, Kyle S. Morrison, Joshua A. Weiner, Margaret E. Ackerman, Vicky Roy, Galit Alter, Guido Ferrari, David C. Montefiori, Georgia D. Tomaras, Sheetal Sawant, Nicole L. Yates, Chris Gast, Sylvain Fleury, Ruth M. Ruprecht

**Affiliations:** ^1^Texas Biomedical Research Institute, San Antonio, TX, United States; ^2^Department of Pulmonary Medicine, Bern University Hospital, University of Bern, Bern, Switzerland; ^3^Mymetics SA, Epalinges, Switzerland; ^4^New Iberia Research Center, University of Louisiana at Lafayette, Lafayette, LA, United States; ^5^Department of Biology, University of Louisiana at Lafayette, Lafayette, LA, United States; ^6^Department of Microbiology and Immunology, Geisel School of Medicine at Dartmouth, Hanover, NH, United States; ^7^Thayer School of Engineering, Dartmouth College, Hanover, NH, United States; ^8^Ragon Institute of Massachusetts General Hospital (MGH), Massachusetts Institute of Technology (MIT) and Harvard, Cambridge, MA, United States; ^9^Massachusetts Consortium on Pathogen Readiness, Boston, MA, United States; ^10^Department of Surgery, Duke University, Durham, NC, United States; ^11^Duke Human Vaccine Institute, Duke University School of Medicine, Durham, NC, United States; ^12^Department of Molecular Genetics and Microbiology, Duke University, Durham, NC, United States; ^13^Department of Immunology, Duke University, Durham, NC, United States; ^14^Fred Hutchinson Cancer Research Center, Seattle, WA, United States

**Keywords:** HIV-1 gp41, virosomes, virosomal vaccine, intramuscular prime/intranasal boost vaccination, Indian-origin rhesus macaque model, SHIV, intravaginal challenge, mucosal immunity

## Abstract

A virosomal vaccine inducing systemic/mucosal anti-HIV-1 gp41 IgG/IgA had previously protected Chinese-origin rhesus macaques (RMs) against vaginal SHIV_SF162P3_ challenges. Here, we assessed its efficacy in Indian-origin RMs by intramuscular priming/intranasal boosting (n=12/group). Group K received virosome-P1-peptide alone (harboring the Membrane Proximal External Region), Group L combined virosome-rgp41 plus virosome-P1, and Group M placebo virosomes. Vaccination induced plasma binding but no neutralizing antibodies. Five weeks after boosting, all RMs were challenged intravaginally with low-dose SHIV_SF162P3_ until persistent systemic infection developed. After SHIV challenge #7, six controls were persistently infected versus only one Group L animal (vaccine efficacy 87%; *P*=0.0319); Group K was not protected. After a 50% SHIV dose increase starting with challenge #8, protection in Group L was lost. Plasmas/sera were analyzed for IgG phenotypes and effector functions; the former revealed that protection in Group L was significantly associated with increased binding to FcγR2/3(A/B) across several time-points, as were some IgG measurements. Vaginal washes contained low-level anti-gp41 IgGs and IgAs, representing a 1-to-5-fold excess over the SHIV inoculum’s gp41 content, possibly explaining loss of protection after the increase in challenge-virus dose. Virosomal gp41-vaccine efficacy was confirmed during the initial seven SHIV challenges in Indian-origin RMs when the SHIV inoculum had at least 100-fold more HIV RNA than acutely infected men’s semen. Vaccine protection by virosome-induced IgG and IgA parallels the cooperation between systemically administered IgG1 and mucosally applied dimeric IgA2 monoclonal antibodies that as single-agents provided no/low protection – but when combined, prevented mucosal SHIV transmission in all passively immunized RMs.

## Introduction

The first cases of unexplained acquired immunodeficiency syndrome in young individuals, later termed AIDS, were described approximately 40 years ago, and the causative agent, HIV-1, was discovered in 1983 (1). Since the beginning of the AIDS pandemic, HIV has infected ~75.7 million individuals and caused 32.7 million deaths (UNAIDS). Approximately 90% of all new HIV infections are the result of mucosal exposures, including sexual and perinatal transmission events. During HIV sexual transmission, the genital and rectal tissues are the main virus entry points where primary infection foci are established, from which the virus spreads into the intestinal tract and other host organs. Newly transmitted HIV strains almost exclusively use CCR5 as coreceptor (R5 strains) and are relatively difficult to neutralize (tier 2 strains). In general, a newly infected individual harbors one predominant strain initially, the so-called transmitted founder virus. This is the case even if the source person harbors a multitude of HIV quasi-species.

Despite intense efforts by multiple groups, there is no safe and effective vaccine against HIV/AIDS. A number of Phase 3 clinical trials showed lack of efficacy (2–4), with the one exception of the RV144 trial that showed a 31.2% reduction in the risk of HIV acquisition among the vaccinees, compared to individuals given placebo (5). Most vaccine strategies involving HIV envelope immunogens focused on gp120, gp140, or gp160 and did not include analyses of mucosal immune responses in trials performed in nonhuman primate (NHP) models or humans. Notable exceptions include studies performed by the team of Robert-Guroff (6, 7) who tested mucosal delivery of vaccine antigens through either the intranasal or intratracheal routes [reviewed in (8)].

Furthermore, subunit vaccine administration has often involved a single parenteral route (9–11) or sometimes by single mucosal administration (12, 13), but rarely involved combined mucosal and intramuscular (i.m.) immunizations as was done with virosomal vaccines (14, 15). Our team and others have postulated that an effective HIV/AIDS vaccine must be capable of eliciting both systemic and mucosal immune protection for maximal protection of different mucosal portals of entry. However, due to the compartmentalized mucosal and systemic immune systems, the induction of strong immune responses in various local and distant mucosal tissues and in the systemic compartment is challenging. The traditional parenteral immunization involving the i.m. or subcutaneous (s.c.) routes can elicit circulating B and T cells that generally remain mostly in the periphery.

**Figure 1:**
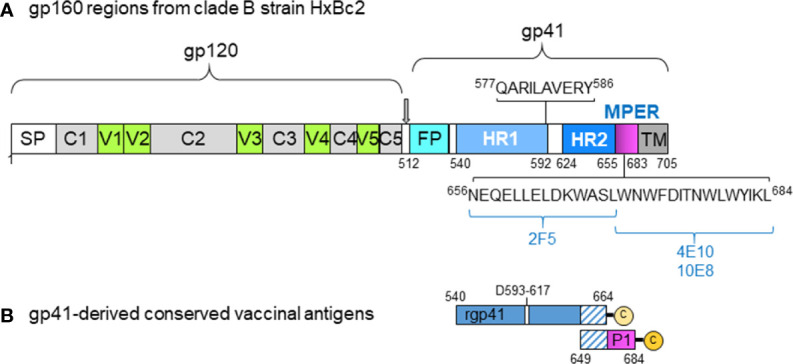
HIV gp41-derived antigens used with virosomes. **(A)** Scheme illustrating the various regions of HIV gp120 and gp41 with the signal peptide (SP), variable regions #1 to #5 (V1-V5), conserved domains #1 to #5 (C1-C5), gp41 fusion peptide (FP), the two helix regions 1 and 2 (HR1 and HR2), the membrane-proximal external region (MPER), and the transmembrane domain (TM). The numbers indicated correspond to the amino acid position in the HxBc2 gp160 sequence, with some of the key neutralizing gp41 epitope sequences (QARILAVERY, 2F5, 4E10, 10E8) to show their location. **(B)** The recombinant gp41 antigen (rpg41) covers the amino acid sequence 540-664 with a deletion from 593-617, followed by leucine and glutamic residues from the cloning site and a 4-histidine tag sequence ending with a C-terminal cysteine for lipidation. The last 16 rgp41 residues on the C-terminal end (residues 649-664) overlap with the first 16 amino terminal residues of the P1 peptide. Of note, the originally described P1 sequence covers the gp41 residues 649-683, and it was subsequently modified by adding the natural leucine residue (649-684) on the C-terminal end, followed by serine and cysteine residues to improve peptide solubility, stability, upscaling as well as allowing lipidation. This modified P1 sequence and the rgp41, respectively, were anchored onto separate virosomes and used for nonhuman primate studies.

**Figure 2:**
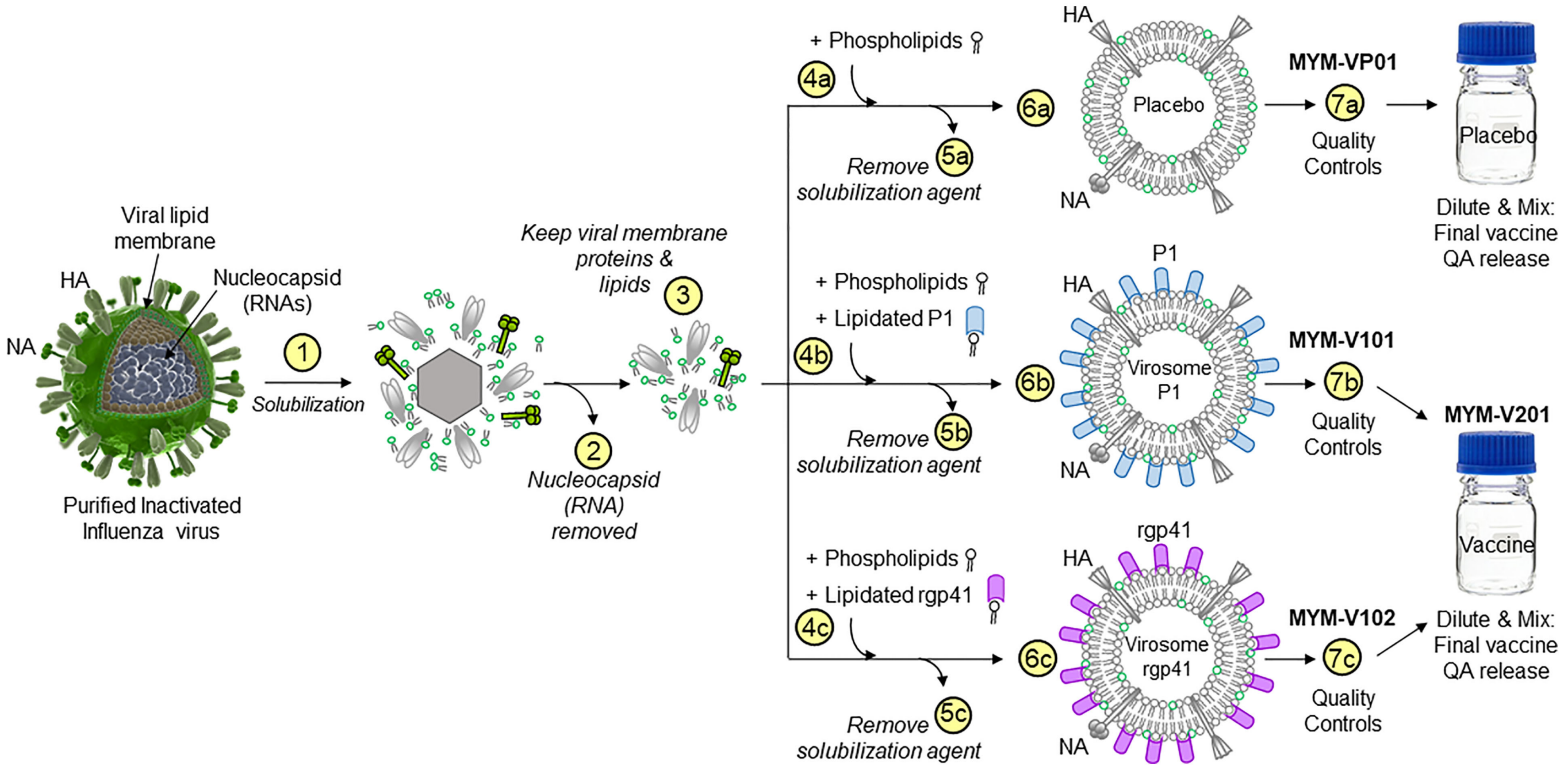
Production schema to generate HIV-1 gp41 virosomes. The production of unadjuvanted placebo virosomes (MYM-VP01), as well as unadjuvanted virosome-P1 (MYM-V101) and virosome-rgp41 (MYM-V102) is based on components derived from influenza virus membranes. Step 1, inactivated influenza A/H1N1 is solubilized with detergent; Step 2, nucleocapsids are discarded; Step 3, the viral membrane lipids with the native influenza hemagglutinin (HA) and neuraminidase (NA) are recovered and used as carriers for vaccinal antigens. The following steps are specific to each virosomal vaccine: Step 4a for placebo virosomes; only synthetic phospholipids are mixed with the influenza virus-derived components, while the antigen P1 (Step 4b, blue rods) or rgp41 (Step 4c, pink rods) is mixed with synthetic phospholipids. During gradual removal of the detergent in Steps 5a-5c, placebo virosomes (6a), virosome-P1 (6b), and virosome-rgp41 (6c) are gradually assembled *in vitro*. To generate the HIV-1 liquid virosomal vaccine, virosome-P1 (MYM-V101) and virosome-rgp41 (MYM-V102) are then diluted, combined, and mixed to achieve the target antigen concentration of the final HIV-1 vaccine MYM-V201. Quality control is performed to verify that values for particle size, particle population homogeneity, antigen and HA content are within the predefined specificities.

The approach of an HIV vaccine immunization regimen combining the classical i.m. immunization route with mucosal boosting using the intranasal (i.n.) route was proposed as an alternative to induce systemic as well as mucosal anti-HIV immunity. This notably different vaccine strategy was evaluated with unadjuvanted influenza virus-based virosomes displaying HIV gp41 antigens; vaccine-induced systemic and mucosal antibody (Ab) responses were assessed in Chinese-origin rhesus macaques (RMs) that were immunized *via* two routes followed by intravaginal simian-human immunodeficiency virus (SHIV) challenges (14). The gp41 antigens were derived from Env regions highly conserved across multiple HIV clades and strains **(****Figure 1****)**. These virosomes are lipid-based particles reconstituted *in vitro* from influenza viruses but devoid of nucleic acids and thus non-infectious (**Figure 2**). One population of virosomes was assembled to display on their surface Peptide 1 (P1), an extended version of the Membrane Proximal External Region (MPER) of HIV gp41, to generate virosome-P1. Another virosome population displayed recombinant, truncated gp41 (virosome-rgp41); rgp41 is devoid of the immunodominant region that contains the KLIC motif as well as other domains homologous to human host proteins. The combined vaccine preparation that consists of virosome-P1 plus virosome-rgp41, is termed MYM-V201 (**Figure 2****)**.

The initial study in Chinese-origin RMs evaluated only the combination of virosome-P1 plus virosome-rgp41 (but not single-agent virosomes). Control RMs received placebo virosomes devoid of HIV gp41 antigens (14), and two groups of vaccinees were given either four i.m. immunizations or two i.m. immunizations followed by two i.n. boosts, respectively. All animals were challenged intravaginally by repeated low-dose exposures to SHIV_SF162P3_, an R5-tropic, tier 2, clade B strain; the challenge SHIV encoded a heterologous gp41 sequence compared to that in the immunogens. Priming *via* the i.m. route followed by i.n. boosting was remarkably effective: 100% of the animals were protected and did not seroconvert to SIV Gag, a viral protein absent in the vaccine. However, protection was not sterile as some animals had low-level viral RNA blips just at the limit of detection (14).

Attempts to identify correlates of protection were made in this initial study (14); no links were found with any Ab parameters from the systemic compartment, and no neutralizing antibodies (nAbs) were detected in serum samples. However, protection was linked to mucosal Ab characteristics; there was a correlation with neutralizing IgG found in vaginal fluids as well as IgG-mediated antibody-dependent cellular cytotoxicity (ADCC) activity, again only from vaginal IgGs. Furthermore, vaginal IgAs from vaccinated animals blocked virus transcytosis in cell-culture assays.

The study summarized above stands out also because it was performed in Chinese-origin RMs – as opposed to the standard subspecies, Indian-origin RMs. Most SHIV strains have been adapted to Indian RMs, although they replicate in animals of the Chinese subspecies. The pathogenicity of some SIV and SHIV strains differs between the two subspecies, predominantly because Indian-origin RMs have been used to optimize the replicative capacity and virulence of primate immunodeficiency viruses used for vaccine challenge studies. In addition, since most vaccine efficacy studies have made use of Indian RMs, this allows for estimation of relative vaccine efficacy in comparison to other vaccine approaches.

Here we report a repeat study performed in Indian-origin RMs conducted at a different animal facility with the combination of unadjuvanted virosomes-P1 plus virosomes-rgp41, termed MYM-V201, using repeat low-dose intravaginal challenges. We included an additional group to evaluate animals vaccinated with the single-agent unadjuvanted virosomes-P1. The rationale for including the latter group is the successful conclusion of a Phase 1 clinical study with virosomes-P1 in low-risk women (15), where this vaccine was safe and immunogenic. However, no efficacy data existed from NHP studies regarding virosome-P1 as single immunogen.

The current study demonstrated significant protection for the combination of virosomes-P1 plus virosomes-rgp41 that – depending on the read-out – ranged from 78% to 87% during the first SHIV challenge phase, i.e., challenges #1 to #7, up to the day of but not including challenge #8 (termed Challenge Phase I). However, when the SHIV inoculum was increased by 50% as in the earlier study in Chinese RMs (14), protection was lost. Single-agent virosomes-P1 showed no efficacy throughout both SHIV challenge phases. We conclude that the combination of the two HIV gp41 virosomes, virosomes-P1 plus virosomes-rgp41, was safe, immunogenic, and effective as long as the intravaginal SHIV inoculum was within a 100-fold excess over the HIV RNA levels found in the semen of acutely infected men (16), or within a 70,000-fold excess of the median semen viral RNA content men who were part of HIV discordant heterosexual couples (17).

## Materials and Methods

Three groups of 12 Indian-origin female rhesus macaques (RMs; *Macaca mulatta)* were enrolled; the animals were housed at the Southwest National Primate Research Center (SNPRC), San Antonio, Texas, USA. Approval for all procedures was received from the Institutional Animal and Care and Use Committee of the Texas Biomedical Research Institute. The RMs were maintained according to the guidelines established by the Animal Welfare Act and the NIH Guide for the Care and Use of Laboratory Animals, with protocols approved by the local ethical committee.

All RMs were negative for Mamu B*08 and Mamu B*17 alleles. Prior to enrollment, peripheral blood mononuclear cells (PBMC) of all RMs were tested for their ability to support the replication of challenge virus, SHIV_SF162P3_ (18, 19); p27 levels in culture supernatants were measured using the SIV p27 Antigen Capture Assay kit (ABL Inc.). Only RMs able to support challenge SHIV replication were selected for the study.

### Animal Randomization and Enrollment

In order to avoid imbalance in covariates likely to influence outcomes that may result from an unfortunate instance of simple random sampling, the method of rerandomization (20) was employed to randomly assign RMs to four groups: three experimental groups of 12 animals (Groups K, L and M) as well as a titration group (n=6) to confirm the infectious challenge virus dose (a second titration group (n=6) was enrolled later). Covariates to be balanced across groups were age, weight, number of males with which each animal was previously co-housed, and number of offspring **(****Table 1****)**. For each of 500,000 completely random allocations to study group, the Mahalanobis distance (21) was computed as the scaled difference between vectors formed in such a way that all pairwise differences in covariates between groups were computed for a given randomization instance, with the scaling matrix block-diagonal, with each block obtained as the generalized inverse of the covariance matrix of the variables of interest from all animals available for randomization. A cutoff value was chosen such that ~5% of the best (most balanced) potential randomizations were eligible to be selected for use. The subset was further limited by requiring no cells in the contingency table of FcR3A by group to be 0, no group would have fewer than 4 of the individuals previously co-housed with no males, and no group would have fewer than two Mamu A*01-positive animals, and two animals which had already been designated for the titration group were always assigned to this group. In order to enable randomization hypothesis tests across the three study groups, the final set of eligible randomizations to be used were then obtained *via* implementation of the same method but excluding those randomized to the titration group, by retaining those with the dissimilarity metric less than the median of those used in the first round (**Table 1**).

**Table 1:** Distribution of demographic factors across Indian RMs of the different study groups.

		Group K: virosomes-P1 (n=12)	Group L: virosomes-P1 + virosomes-gp41 (n=12)	Group M: Control (n=12)	Titration Group (n=6)	p-value^1^
Age, mean years (SD)		7.9 (3.8)	9.6 (5.2)	8.3 (5.0)	7.8 (5.1)	0.960
Weight, mean kg (SD)		7.0 (1.8)	7.6 (2.4)	6.3 (1.3)	7.1 (2.8)	0.671
# Offspring, mean (SD)		1.8 (1.9)	1.4 (1.6)	2.0 (2.3)	0.5 (0.8)	0.559
# Males mated with (SD)		1.0 (1.0)	1.1 (1.0)	0.9 (1.0)	0.8 (1.3)	0.874
Genetic screening:						
FcγR3A	Bad	6 (50%)	4 (33%)	5 (42%)	1 (17%)	0.809
	Good	2 (17%)	1 (8%)	1 (8%)	1 (17%)	
	Intermediate	4 (33%)	7 (58%)	6 (33%)	4 (67%)	
Mamu A*01	Negative	8 (67%)	10 (83%)	8 (67%)	4 (67%)	0.815
	Positive	4 (33%)	2 (17%)	4 (33%)	2 (33%)	

^1^Kruskal-Wallis test for continuous variables, Fisher Exact test for categorical variables.

### Liquid Virosome Manufacturing

HIV-derived antigens inserted into the virosome membrane were previously described (14, 15, 22, 23). The synthetic P1 lipopeptide (amino acid sequence 649-683 of gp41) was produced by Bachem AG (Bubendorf, Switzerland). The recombinant gp41-derived antigen (amino acid 540-664 with a deletion of 25 amino acids from 593-617, plus a C-terminal 4xHis-tag for purification followed by a free cysteine) was expressed in *E. coli* and purified as trimers under non-denaturing conditions by PX’Therapeutics (Grenoble, France). Lipidation of the C-terminal cysteine of the rgp41 to 1,2-Dipalmitoyl-sn-glycero-3-phosphoethanolamine-N-[4-(p-maleinimidomethyl)cyclohexane-carboxamide]) (N-MCC-DPPE, Corden Pharma, Liestal, Switzerland) allowed antigen anchorage into the virosome lipid membrane produced under liquid form, as described (14). The antigens were dissolved in 100 mM octaethylene glycol monododecyl ether (OEG, Sigma, Buchs, Switzerland) prepared in HN buffer (50 mM HEPES pH 7.4, 142 mM NaCl) and added to the virosome excipients during manufacturing. The final HIV-1 liquid virosomal vaccine MYM-V201 for the i.m. route (0.5 ml) contained 85 µg/ml hemagglutinin (HA), 90 µg/ml P1, 130 µg/ml rgp41, and was supplied in HN buffer pH 7.4. For the i.n. formulation, a similar dose was delivered but in 0.2 ml volume (0.1 ml per nostril) using the BD Accuspray. Nanoparticle tracking analysis (NTA) for the virosome particle size was performed on a Malvern NS300 instrument. Dynamic light scattering (DLS) to determine virosome population homogeneity based on the polydispersity index (PDI) was performed on a Malvern Zetasizer Nano. Microbiological quality was determined according to E.P. section 5.1.4. Absence of specific microorganisms was demonstrated according to E.P. section 2.6.13 - *Pseudomonas aeruginosa* and *Staphylococcus aureus*.

### Immunizations

**Figure 3:**
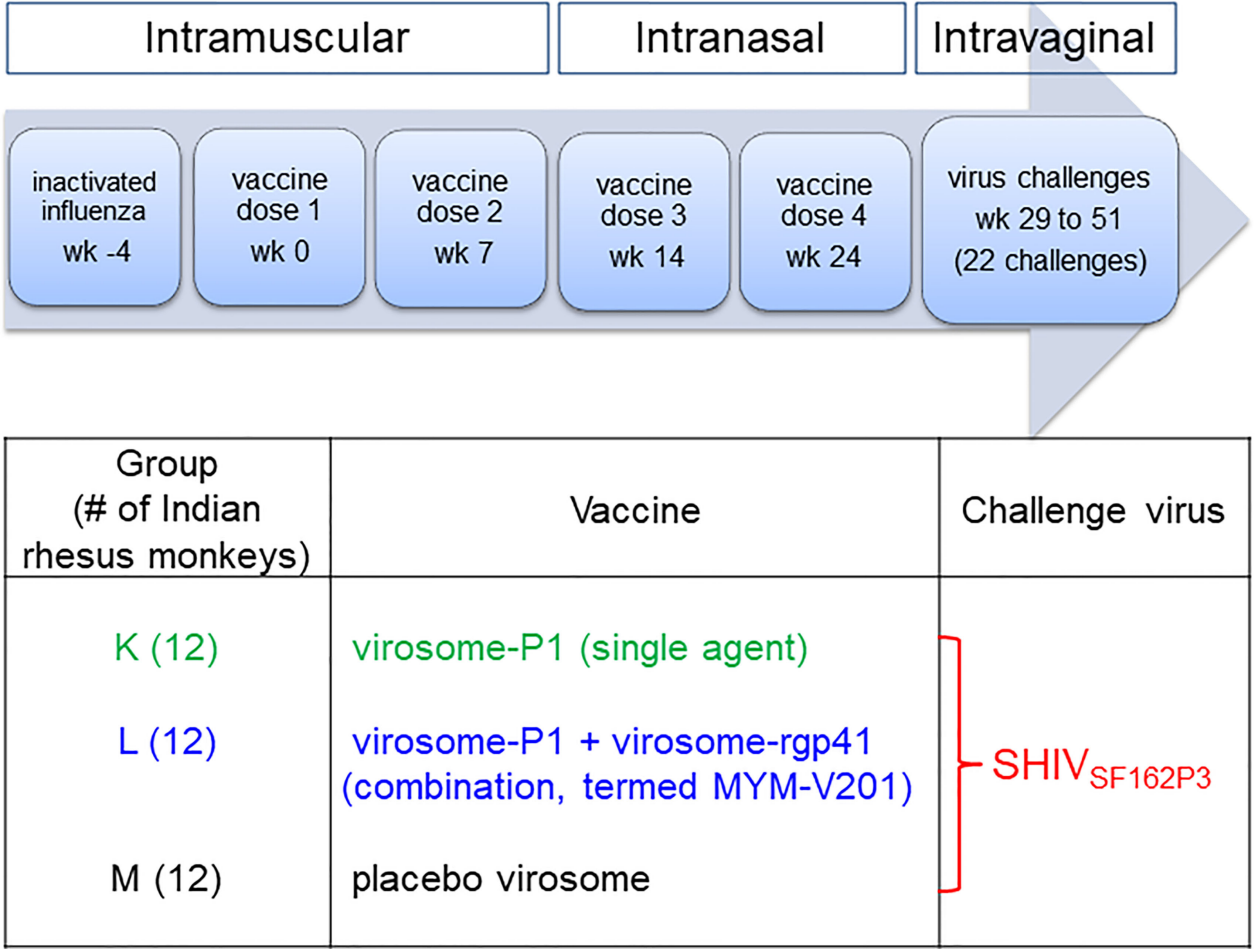
Study design and timeline for vaccine administration and virus challenges. For three groups of 12 Indian rhesus macaques, inactivated influenza virus was given intramuscularly 4 weeks (week -4) before the first intramuscular vaccine dose (week 0), which was followed by the second intramuscular vaccine dose at week 7. The third and fourth vaccine doses were given intranasally at weeks 14 and 24, respectively. Intravaginal SHIV_SF162P3_ virus challenges were performed from week 29 to week 51. The challenges were stopped after a given animal became persistently viremic (>10,000 copies/ml) or after 22 challenges. During the vaccination phase, the study was performed blinded. For Groups K and L, blinding was maintained throughout the entire study; Group M animals were recognized as controls after immunogenicity analyses for anti-P1 peptide antibody reactivity prior to starting the SHIV_SF162P3_ challenges.

Four weeks before the first virosome administration, Groups K, L, and M were given 10 μg of HA from inactivated influenza virus (strain A/Brisbane/59/2007 H1N1) to mimic the pre-existing natural or vaccine-induced anti-influenza immunity in humans. This influenza virus was propagated in the allantoic cavity of embryonated eggs and purified as described (24). The vaccine doses were 45 µg of P1 and 65 µg of rgp41 for each of the i.m. and i.n. immunizations. The immunization schedule for the RMs in Groups K, L, and M is depicted in **Figure 3**; all investigators and veterinary staff were blinded during the vaccinations.

### Virus

The challenge virus, SHIV_SF162P3_ (a tier 2, R5 clade B strain (18, 19), was kindly provided by Dr. Nancy Miller, NIAID (NIH-AIDS Research and Reference Reagent Program (NIH-ARRRP)); the genome of this SHIV contains the *env*, *tat*, *rev*, and *vpu* genes of HIV-1 SF162 inserted into the backbone of the pathogenic SIVmac239. The stock was grown in RM PBMC; it had a p27 concentration of 183 ng/ml and 3.5 x 10^5^ 50% tissue culture infectious doses (TCID_50_)/ml as measured in TZM-bl cells. Using ELISA, the total gp41 concentration present in the virus stock was found to be 11 ng/ml. Therefore, the gp41 concentration was 0.13 ng/ml for the first seven challenges (virus stock diluted 1/86 to obtain 15 TCID_50_), and 0.19 ng/ml for the subsequent challenges (virus stock diluted 1/57 to obtain 22 TCID_50_ as measured in RM PBMC); these gp41 concentrations were used to estimate the approximate molar ratio of gp41 antigen molecules to specific Ab molecules in vaginal fluid.

The SHIV_SF162P3_ stock used in the Chinese RM study (14) was no longer available for our study in Indian RMs. Hence, the new virus stock was tested in RMs by intravaginal titration experiments, as *in vivo* infectivity for a given challenge route is the gold standard. Our most important criterion was to achieve persistent viral infection using approximately the same number of intravaginal challenges, at the same time intervals, as in the original study (14). We enrolled a total of 12 Indian RMs in the titration to extrapolate the new SHIV_SF162P3_ stock’s intravaginal challenge dose. The latter was also expressed as TCID_50_ (measured in Indian RM PBMC) to show that its infectivity was in the same order of magnitude as that of the stock used previously in Chinese RMs. Importantly, TCID_50_ measurements are only operational terms but not absolute units of measure. TCID_50_ values for any given stock vary from lab to lab for the following reasons: i) PBMC collected from RM donors are virus target cells that cannot be standardized given outbred nature of RMs; ii) TCID_50_ data also depend on the cell culture conditions and individual laboratory procedures, and iii) assay readout sensitivity can vary greatly. Consequently, TCID_50_ values measured in primary RM PBMC give only order-of-magnitude information rather than absolute values.

### Intravaginal SHIV Challenges

Following similar timeline and virus TCID_50_ doses as described in the Chinese RM study (14), five weeks after the second i.n. boost (week 29), all animals were inoculated intravaginally with low-dose virus once or twice per week, with 15 TCID_50_ of the heterologous SHIV_SF162P3_ for the first 7 challenges and 22 TCID_50_ for challenges 8 to 22. The veterinary procedures outlined in Chenine et al. (25) were used; RMs undergoing vaginal SHIV challenges were not treated with hormones.

### Plasma Viral RNA Levels

Plasma vRNA was isolated by QiaAmp Viral RNA Mini-Kits (Qiagen, Germantown, MD, USA); vRNA levels were measured by quantitative reverse-transcriptase polymerase chain reaction (RT-PCR) for SIV *gag* sequences (26, 27). Assay sensitivity was 50 copies/ml.

### Statistical Methodology to Assess Vaccine Efficacy

Time-to-event methodology was used to evaluate the occurrence of primary and secondary endpoints. As the intravaginal challenges were not equally spaced, the time variable is presented and analyzed in units of study days, rather than number of challenges. The endpoints analyzed included: time to first viremia, defined as the first observation of viral load >50 copies/ml; time to peak viremia, using the study day of observed peak viral load; and time to persistent systemic infection (PSI), in which the endpoint is reached when viral RNA is ≥10,000 copies/ml. As the challenge dose was increased from the 8^th^ challenge onward (Challenge Phase II), results are presented overall (Challenge Phases I plus II) and also with endpoints censored on day 32, the date of the 8^th^ challenge (Challenge Phase I).

Randomization tests were used to compare endpoints across group. The *survival* (28) and *interval* (29) packages for R statistical software were used to compute the corresponding log-rank test statistic for each eligible randomization instance, with p-values obtained as the number of test statistics exceeding the value of the statistic for the actual randomization. As a sensitivity evaluation of the randomization test method, the standard log-rank p-value obtained from the null distribution, which assumes random allocation, rather than the restricted allocation described above, was also computed. Vaccine efficacy is computed as one minus the ratio of infection rates (vaccinated vs control group), with supportive confidence intervals obtained by the exact conditional binomial method, assuming Poisson distribution of endpoints within each group (30). No adjustment for multiplicity was done.

### ELISA Ab Assays

HIV-1 gp41-derived antigens for ELISA plates. The P1 peptide powder stock was dissolved first in hexafluoroisopropanol prior adding protease-free sterile water for obtaining a solution stock at 0.7 mg/ml in 10% hexafluoroisopropanol. P1 peptide was then diluted at 2 µg/ml. The frozen rgp41 stock was thawed and diluted with cold phosphate-buffered saline (PBS) pH 7.4 to obtain a solution at 2 µg/ml.

To coat 96-well MaxiSorp flat bottom plates (Nunc), 0.1 ml of freshly prepared antigen solution was added to wells (0.2 µg/well). Plates were wrapped into aluminum foil and incubated 16 h at 4°C. The next day, plates were washed four times with 300 µl/well with PBS pH 7.4 with Tween 20 at 0.05% (PBST) prior adding 300 µl/well of casein as blocking reagent (Abcam, ab 171535), followed by a 2 h incubation at 37°C. Plates were then washed four times with 300 µl/well with PBST and 100 µl of diluted sera, positive RM serum controls, and human monoclonal antibodies (mAbs) 2F5 (31) and 98.6 (32), both from NIH-ARRRP were added to the appropriate wells, plates were wrapped in aluminum foil, and incubated for 2 h at 37°C. Plates were then washed 8x with 300 µl/well with PBST and 100 µl of diluted rabbit anti-monkey IgG horse radish peroxidase (HRP) into PBST (Sigma, A2054, dilution according to the supplier’s recommendation) were added to wells and plates wrapped in aluminum foil prior placing them into the incubator at 37°C for 1 h. Wells were then washed 8x with 300 µl/well, followed by the addition of 100 µl of substrate (TMB single solution, Life Technologies) and plates incubated in the dark (wrapped in aluminum foil) at room temperature for 10-15 min or until the color signal was considered strong enough, then the reaction was stopped by the addition of 100 µl/well of 1 M H_2_SO_4_ and the optical density of the reaction was read at 450 nm.

### Neutralization Assays

NAbs were measured as a function of reduction in luciferase (Luc) reporter gene expression after a single round of infection in either TZM-bl cells (33, 34), TZM-bl/FcγRI cells (35) or A3R5 cells (36). TZM-bl cells (also called JC57BL-13) were obtained from the NIH-ARRRP, as contributed by John Kappes and Xiaoyun Wu. This is a HeLa cell clone engineered to express CD4 and CCR5 (37) and to contain integrated reporter genes for firefly luciferase and *E. coli* beta-galactosidase under control of an HIV-1 long terminal repeat (LTR) (38). The cells were engineered by stable transduction to express human FcγRI cells, which made them ultra-sensitive for detecting gp41 MPER-specific nAbs (35).

For assays in TZM-bl and TZM-bl/FcγRI cells, a pre-titrated dose of virus was incubated with serial 3-fold dilutions of heat-inactivated (56°C, 30 min) serum samples in duplicate in a total volume of 150 μl for 1 h at 37°C in 96-well flat-bottom culture plates. Freshly trypsinized cells (10,000 cells in 100 μl of growth medium containing 75 μg/ml DEAE dextran) were added to each well. One set of control wells received cells + virus (virus control) and another set received cells only (background control). After 48 h of incubation, 100 μl of cells was transferred to a 96-well black solid plate (Costar) for measurements of luminescence using the Britelite Luminescence Reporter Gene Assay System (PerkinElmer Life Sciences). Neutralization titers are the dilution (serum/plasma samples) or concentration (mAbs) at which relative luminescence units (RLU) were reduced by 50% or 80% compared to virus control wells after subtraction of background RLUs. Assays in TZM-bl and TZM-bl/FcγR1 cells used a rhesus PBMC-grown stock of SHIV_SF162P3.R157_.

A3R5 (A3.01/CCR5), a derivative of the A3.01 human lymphoblastoid cell line that naturally expresses CD4 and CXCR4, was engineered to express CCR5 (36). The A3R5 assay was performed with Env.IMC.LucR viruses as described (36). Briefly, serum and plasma samples were assayed at 3-fold dilutions ranging from 1:20 to 1:43,740. Neutralization titers are the sample dilution at which RLU were reduced by 50% as compared to RLU in virus-control wells after subtraction of background RLU in cell-control wells. Assays in A3R5 cells used SHIV-SF162P3.LucR infectious molecular clone produced by transfection in 293T cells.

### Antibody-Dependent Cellular Phagocytosis

The ADCP assay was adapted from (39). Briefly, gp140 SHIV_SF162p3_ and the recombinant Mymetics gp41 antigens were biotinylated using sulfo-NHS LC-LC biotin, coupled to yellow-green, fluorescent Neutravidin 1 μm beads (Invitrogen, F8776) for 2 h at 37°C and washed two times in 0.1% bovine serum albumin (BSA) in PBS. Ten μl/well of coupled beads were added to 96-well plates with 10 μl/well of diluted sample for 2 h at 37°C to form immune complexes. After incubation, the immune complexes were spun down, and supernatants were removed. THP-1 cells were added at a concentration of 2.5 x 10^4^ cells/well and incubated for 18 h at 37°C. After incubation, the plates were spun down, the supernatant was removed, and cells were fixed with 4% paraformaldehyde (PFA) for 10 min. Fluorescence was acquired with a Stratedigm 1300EXi cytometer. Phagocytic score was calculated using the following formula: (percentage of FITC+ cells) * (the geometric mean fluorescent intensity (gMFI) of the FITC+ cells)/10,000. A polyclonal HIVIg pool available from the NIH AIDS Reagent Program was used as a positive control. Serum from a human HIV-seronegative donor was used as negative control.

### Antibody-Dependent Neutrophil Phagocytosis

The ADNP assay was adapted from Karsten et al. (40); gp140 SHIV_SF162p3_ and the recombinant Mymetics rgp41 antigens were coupled to beads and immune complexes were formed as described for ADCP. Neutrophils were isolated from fresh whole ACD-anticoagulated blood using EasySep Direct Human Neutrophil Isolation kit (Stem Cell, 19666), resuspended in R10 medium, and added to plates at a concentration of 5x10^4^ cells/well. The plates were incubated for 30 min at 37°C. The neutrophil marker CD66b (Pacific Blue conjugated anti-CD66b; BioLegend, 305112) was used to stain cells. Cells were fixed for 10 min in 4% PFA. Fluorescence was acquired with a Stratedigm 1300EXi cytometer and phagocytic score was calculated as described for ADCP.

### HIV-1-Specific Binding Antibody Multiplex Assay

HIV-1-specific antibodies were measured by HIV-1 Binding Antibody Multiplex Assay (BAMA) for IgG and IgA as described (41–44). For IgA assays, the samples were depleted by Protein G for sensitivity of IgA detection. The following antigens were used for both IgG and IgA assays: Bio-MPR.03 (MPER) (NEQELLELDKWASLWNWFDITNWLWYIR), MYM gp41 (vaccine gp41 from Mymetics), MYM-P1-PE (P1 from Mymetics), SP62 (MPER-2F5 epitope, QQEKNEQELLELDKWASLWN), gp41 (recombinant MN, Immunodiagnostics) and for IgA, two antigens that corresponded with decreased HIV-1 risk in RV144 were included: 00MSA 4076 gp140 (clade A gp140), A1.con.env03 140 (consensus clade A gp140) (45, 46). Assays were run under GCLP compliant conditions, including tracking of positive controls by Levy-Jennings charts using 21CFR Part 11 compliant software. Positive controls included a HIVIG and SHIVIG (DBM5, purified IgG from SHIV-infected RMs kindly provided by Dr. Mario Roederer, Vaccine Research Center). Additional positive controls included 2F5 IgG, 7B2 IgG, 4E10 IgG and purified RM IgA and negative controls included in every assay were blank well control and uncoupled beads. Antibody measurements were acquired on a Bio-Plex instrument (Bio-Rad, Hercules, CA, United States) using 21CFR Part 11 compliant software and the readout is in Mean Fluorescence Intensity (MFI). The preset assay criteria for sample reporting are: coefficient of variation (CV) per duplicate values for each sample were ≤15% and >100 beads counted per sample. The preset positivity criteria were: 1. MFI-blank well-blank bead ≥antigen specific MFI cutoff (95^th^ percentile of W0 or 100 MFI) and 2. MFI-blank well-blank bead > 3X W0 MFI-blank well-blank bead and 3. MFI-blank well > 3X W0 MFI-blank well.

### Fc Array Method

Fc array analysis was performed blinded to group as described (47, 48) to evaluate polyclonal Ab responses. Briefly, plasma was centrifuged for two min at 14.8 x g, then aliquoted and analyzed for binding to a panel of antigens listed in **Supplementary Table 1**. Plasma was diluted 1:1,000 for detection reagents (**Supplementary Table 2**) of tetramerized Fcγ receptors (Source: Duke Protein Production Facility) and anti-rhesus IgG (Source: Southern Biotech). For increased sensitivity, detection reagents C1q (Source: Sigma) and anti-human IgA (Source: Southern Biotech) were collected with a plasma dilution of 1:250, along with a replicate of the anti-rhesus IgG. Data collection was performed using Luminex Exponent version 4.2 software.

### Antibody-Dependent Cellular Cytotoxicity Assay

We utilized the flow-based GranToxiLux (GTL) assay to test the samples. Recombinant HIV-1 Con-S gp140 was used to coat the cells in the GTL assay (49, 50). The cut-off for positivity in the GTL assay was >8% of Granzyme B activity. The recombinant gp140 was chosen because it is the best immunogen to capture anti-Env binding Abs; gp140 was selected to detect the presence of ADCC responses directed against gp120 and gp41 epitopes. We also tested the samples using the Luciferase-based (Luc) ADCC assay against the SHIV_SF162P3.5_ that expresses the Luciferase reporter genes (51). The analysis of the results was conducted after subtracting the background detected with the pre-immunization samples. After background subtraction, results would be considered positive if the percent specific killing is >15%. The ADCC laboratory tested samples collected from the pre-immune and immunized animals at weeks 25, 26, 29, and 33.

### Immuno-PCR Imperacer^®^ for Mucosal Ab Quantification

Imperacer^®^ combines the ELISA-based method with the qPCR technique to amplify the artificial DNA, conjugated to the detecting molecule (52–55). As previously described (23), DNA-labeled P1 and DNA-labeled rgp41 (Chimera proprietary expertise) were used in bridging assays to quantify specific IgG and IgA antibodies and DNA-labeled IgG anti-IgG or anti-IgA to quantify total IgG and IgA antibodies in a sandwich Imperacer assay. This method is very sensitive (lower detection limit for the current bridge assay is ≥ 0.001 ng/ml), specific, and species independent, as it can detect a broad range of antibody concentrations of any isotype and from any animal origin. Real-time PCR signals were converted to approximate antibody concentrations (ng/ml) by analysis against a reference antibody curve. These antibody concentrations were provided only as indicative values.

## Results

### Overall Goal and Study Strategy

The prime goal of the current study was to confirm the strong protection, which had been reported for the efficacy study performed earlier in Chinese RMs (14), in the more widely used Indian RM subspecies. Thus, detailed immunogenicity analyses were contingent on first demonstrating the reproducibility of vaccine protection, and a “go/no-go” decision to initiate intravaginal SHIV challenges involved only a minimal confirmation of vaccine immunogenicity. Since both experimental Groups K and L (**Figure 3****)** had received virosomes displaying the P1 peptide, we measured anti-P1 Ab responses using ELISA. For each animal, plasma samples collected before the first vaccination and at specific time points during and post vaccination were tested. Animals from Groups K and L had measurable anti-P1 Abs compared to autologous pre-immune samples (data not shown); the anti-P1 responses in two of the three groups led us to start the SHIV challenges.

In Group M (given placebo virosomes; **Figure 3**), no anti-P1 Abs were detectable. Only two scientists leading the NHP study were aware that Group M RMs were controls; veterinary staff directly involved in the animal experiments did not know the status of any of the three groups during the ensuing SHIV challenges. Regular viremia levels were assessed immediately to decide whether SHIV challenges should continue for any given RM; the experimental design called for stopping challenges once an RM reached vRNA levels ≥10^4^ copies/ml.

### Vaccine Efficacy During Intravaginal SHIV Challenge Phase I

**Figure 4:**
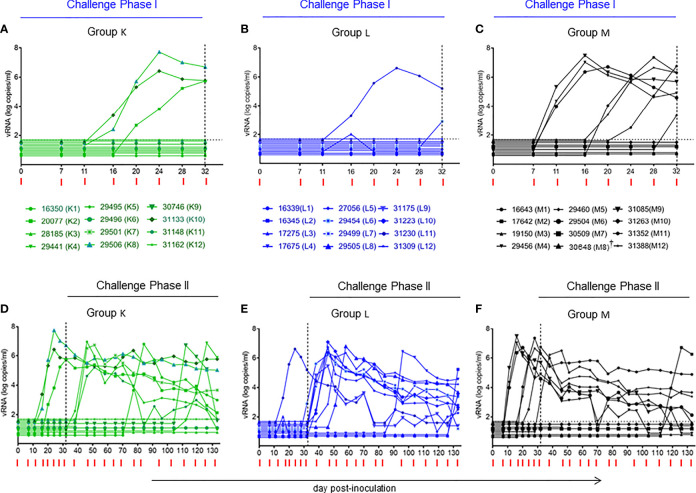
Plasma viral RNA (vRNA) loads after repeated low-dose intravaginal challenges with the tier 2 R5 SHIV_SF162P3_. **(A–C)** Challenge Phase I, comprising the first seven intravaginal challenges with plasma vRNA loads up to day 32, just before challenge #8. **(D–F)** Challenge Phases I (days 0-32) and II (day 32 to end of study). For challenge Phase II, the SHIV_SF162P3_ dose had to be increased by 50% to follow the same experimental strategy used in the initial study in Chinese macaques (14). Horizontal dotted line **(A–F)**, limit of detection 50 vRNA copies/ml (25). Vertical dotted line on day 32 in all panels, start of Challenge Phase II at the increased virus dose. Red ticks, day for each intravaginal SHIV challenge in all panels. ^†^animal 30648 (M8) died of unrelated causes on day 46.

**Figure 5:**
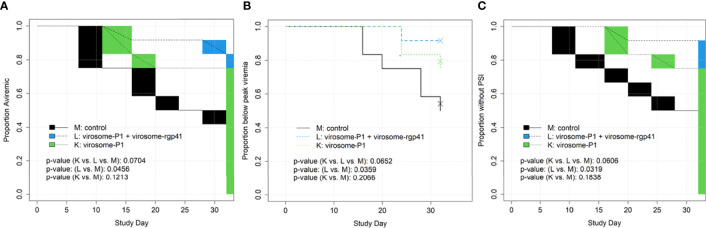
Time to event endpoints, by study day, for SHIV Challenge Phase I **(A–C)**. This included the first 7 challenges and the time up to but not including challenge #8, when the challenge virus dose was increased. Endpoints include **(A)** time to first viremia; **(B)** time to peak viremia; and **(C)** time to persistent systemic infection (PSI). For interval-censored endpoints (time to first viremia, time to PSI), blocks in figures indicate periods with no plasma samples taken, over which the time to event curve is linearly interpolated. There were no significant differences among Groups K, L, and M when the entire study was analyzed (Challenges Phases I and II; for a total of 22 SHIV challenges; data not shown). Statistical analyses were performed by Dr. Chris Gast.

As the primary goal of this current vaccine efficacy study in Indian RMs was to confirm the efficacy observed in Chinese RMs (14), SHIV challenges were performed as in the original study in two phases, starting with 15 TCID_50_ (as assessed in RM PBMC) for the first seven challenges (Challenge Phase I, **Figures 4A–C**), followed by a 50% increase of the inoculum from challenge 8 onwards (Challenge Phase II, **Figures 4D–F**). The results of the plasma vRNA loads for Challenge Phase I up to day 32 are shown in **Figures 4A–C**. During this time span, Groups L and M showed significant differences by three parameters (**Table 2****)**. Using time-to-first viremia, efficacy was 78.4% (p=0.0456); using time-to-peak viremia, efficacy was 85.3% (p=0.0359), and time-to-persistent systemic infection (PSI, defined as vRNA loads ≥10^4^ copies/ml), efficacy was 87% (p=0.0319) (**Figure 5**). In contrast, the analysis of vRNA loads of Group K versus M revealed no protection. We conclude that compared with control Group M, Group L animals had significant vaccine protection during Challenge Phase I; these animals had been vaccinated with the combination of virosomes displaying either P1 or rgp41 (**Figure 3**).

**Table 2:** Vaccine efficacy estimates and comparisons.

Endpoints	Group	Vaccine Efficacy (%)	Global Randomization (*standard*) Test^1^	Randomization test p-value, pairwise comparisons	95% Confidence Interval for Vaccine Efficacy^2^
*Analysis of SHIV Challenge Phases I & II (full duration)*					
Time to first viremia	K vs M	11.6	0.5671 (*0.5611*)	0.8299	(-163.2, 69.7)
	L vs M	-54.6		0.6278	(-335.9, 41.9)
Time to peak viremia	K vs M	10.3	0.8872 (*0.8904*)	0.7601	(-167.3, 69.3)
	L vs M	-21.6		0.9198	(-254.6, 56.8)
Time to PSI	K vs M	12.3	0.7177 (*0.7060*)	0.7327	(-161.2, 70.0)
	L vs M	-37.3		0.8188	(-293.2, 49.7)
*Analysis of SHIV Challenge Phase I (prior to challenge dose escalation)*					
Time to first viremia	K vs M	64.6	0.0704 (*0.0712*)	0.1213	(-55.0, 94.1)
	L vs M	78.4		0.0456*	(-13.5, 97.8)
Time to peak viremia	K vs M	54.9	0.0652 (*0.0610*)	0.2066	(-111.2, 92.7)
	L vs M	85.3		0.0359*	(-21.3, 99.7)
Time to PSI	K vs M	58.6	0.0606 (*0.0642*)	0.1838	(-93.6, 93.3)
	L vs M	87.0		0.0319*	(-7.5, 99.7)

^1^Primary inference drawn from randomization tests; standard p-value included for sensitivity analysis of global test for difference across all groups.

^2^Confidence intervals do not account for constrained randomization and are to be considered supplementary.

*Value <0.05.

### Loss of Vaccine Efficacy During Intravaginal SHIV Challenge Phase II

**Figure 6:**
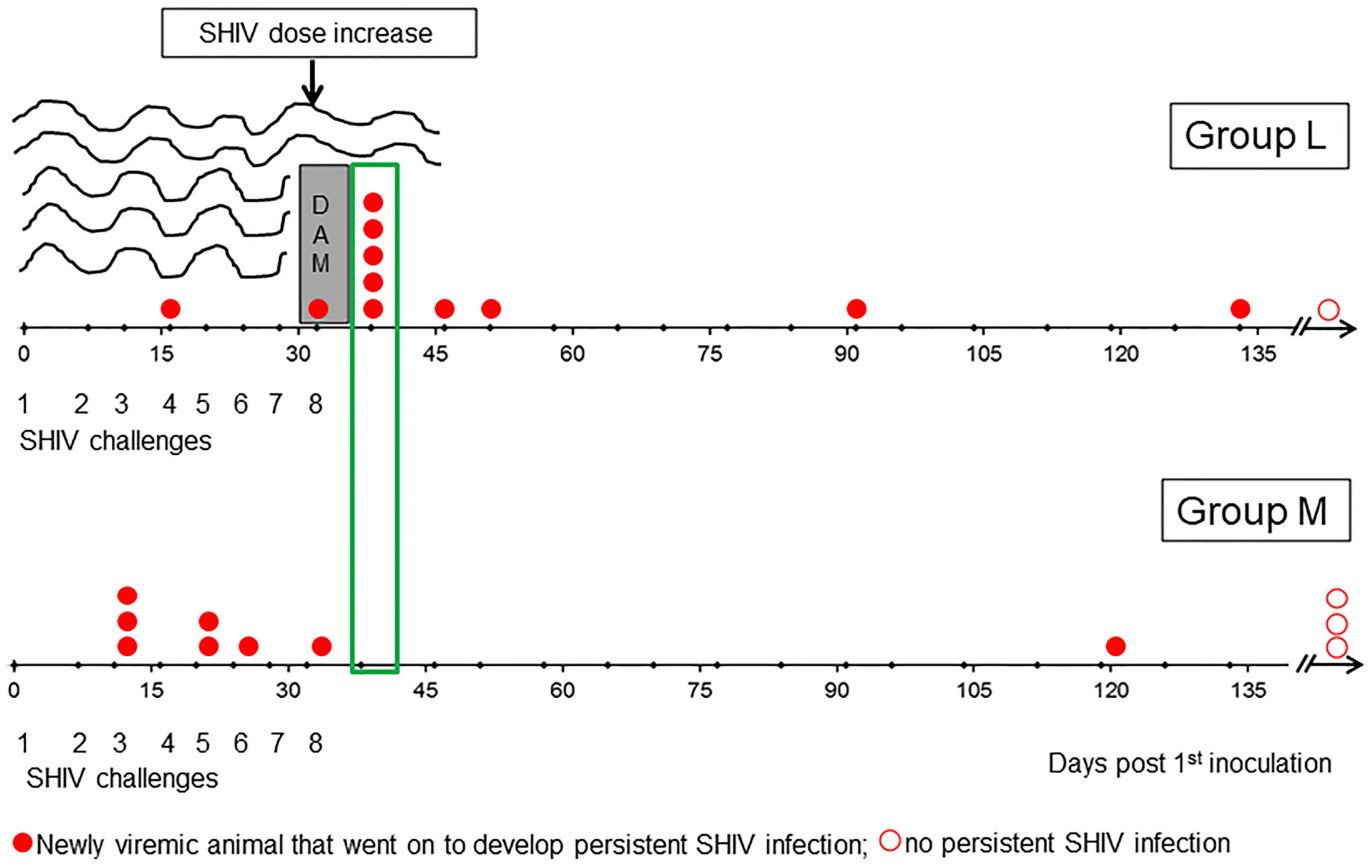
Sudden loss of protection in Group L one week after challenge #8 when the virus dose was increased by 50%. On day 38, five Group L animals lost protection (green box). We hypothesize that vaccine-induced mucosal antibodies were quite protective during Challenge Phase I, when only two breakthrough infections occurred. However, the additional antigen load in the 50% higher challenge virus dose overwhelmed vaccine protection in a large fraction of Group L animals immediately after the dose escalation – akin to flood water overtopping the dam, leading to flash floods and inundation. The implication of this “dam” hypothesis is that the immunogenicity of the virosome platform may need to be increased to boost both mucosal and systemic immune defenses for facing the virus doses administered during experimental intravaginal SHIV challenges in primate models estimated to exceed the HIV-1 inocula passed between infected men to their female partners (please see text).

Following the blueprint of the study in Chinese RMs (14), the SHIV inoculum had to be increased by 50% from intravaginal challenge #8 onwards. One week later, five previously aviremic RMs in Group L became infected and progressed to PSI (**Figure 4E**; red dots in green box, **Figure 6**). The protection seen during Challenge Phase I was no longer seen, and Group K versus Group M again showed no evidence of protection. An in-depth statistical analysis of both Challenges Phase I and II can be found in **Table 2**.

The abrupt loss of protection in Group L immediately following exposure to the higher challenge virus dose implies a threshold effect, according to which vaccine-induced host immune defenses were able to hold the incoming virus at bay as long as the inoculum was not overpowering. Essentially, the situation from challenge #8 onwards is akin to a flash flood overcoming protection provided by a dam (**Figure 6**; please see Discussion). Nevertheless, our analysis is focused on Challenge Phase I in an attempt to identify and describe any protective immune responses observed to be associated with the protection that was evident only in Phase I; importantly, our primary analysis was not limited to this part of the overall study.

### Vaccine-Induced Systemic Antibody Responses in Group L

**Figure 7:**
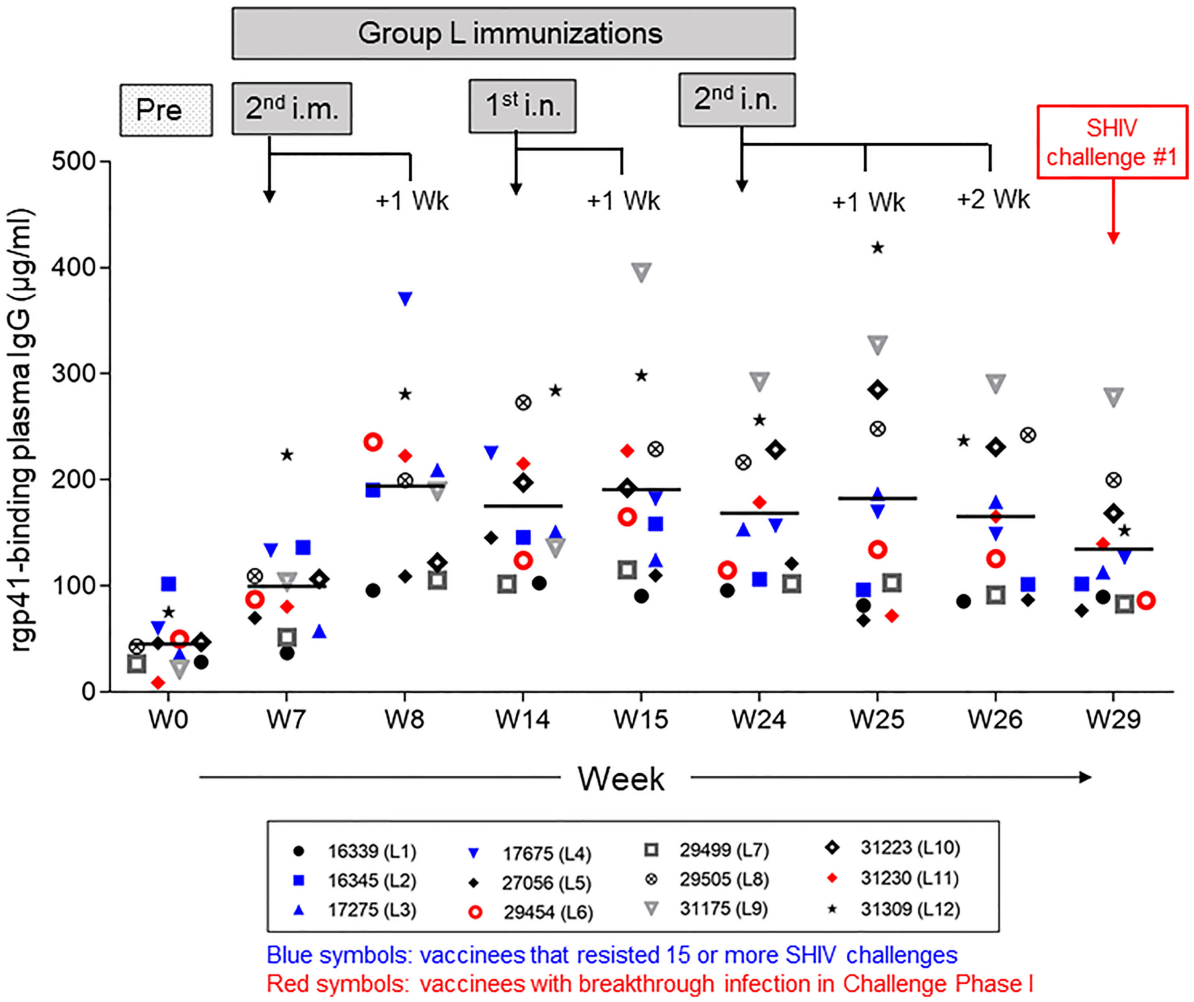
Vaccine-induced anti-HIV-1 gp41 plasma IgG responses in Group L measured by ELISA with rgp41. The timeline for the intramuscular (i.m.) and intranasal (i.n.) immunizations is given in **Figure 3**. Plasma samples collected at the time points indicated were assessed for each individual vaccinee in Group L. Data for the two animals with early breakthrough infection during Challenge Phase I are indicated in red symbols; data for vaccinees that remained aviremic during the first 15 challenges or longer are indicated in blue symbols. No clear pattern emerged for either subset of vaccine recipients.

Given the significant protection seen in Group L during Challenge Phase I, we decided to proceed with analyzing the vaccine-induced antibody responses over time, including pre-immunization, during the i.m. and i.n. vaccine administrations, and at week 29, the day of the first virus exposure (**Figure 7****)**. Individual data points reflect values of each experimental RM. Animals represented by red symbols had breakthrough infections during Challenge Phase I; their anti-rgp41 IgG responses revealed no obvious link with the SHIV challenge outcome.

**Figure 8:**
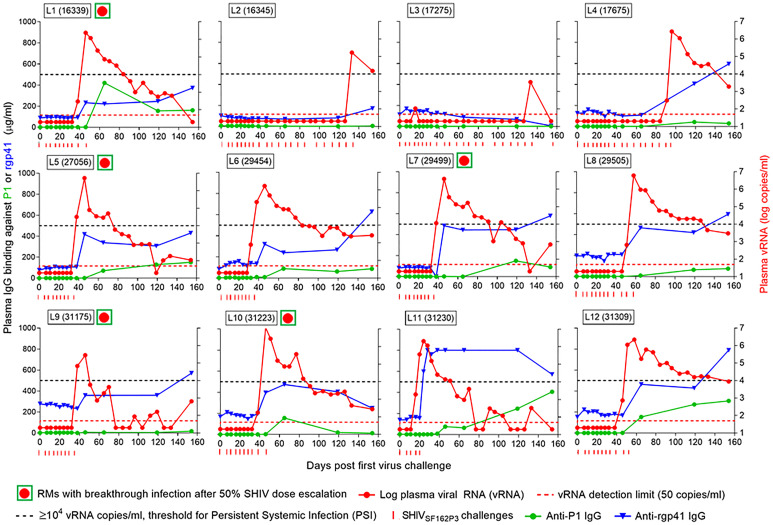
Antigen-specific IgG concentration in plasma samples and viral RNA (vRNA) loads over time. Each panel represents data for one rhesus macaque of Group L that had been vaccinated with the combination of virosome-P1 + virosome-rgp41. Red circles in green squares, RMs with breakthrough infection 6 days after increasing the SHIV challenge dose by 50% on day 32 (challenge #8; please see ). Red dotted line, limit of detection for vRNA [50 copies/ml (25)]. Black dotted line, ≥ 10^4^ plasma vRNA copies/ml, threshold for Persistent Systemic Infection (PSI). Red ticks, time points at which each animal underwent SHIV_SF162P3_ challenges. Once vRNA levels were ≥10^4^ copies/ml, no further virus challenges were administered. The number of SHIV_SF162P3_ challenges thus varied for each animal. Immediately following challenge #8 at the 50% higher virus dose, many Group L animals had breakthrough infections, which were not linked to decreases in anti-P1 or anti-rgp41 plasma IgG levels.

Next, we sought to determine whether the sudden loss of protection seen in Group L vaccinees might be due to decreases in vaccine-induced, antigen-specific Ab levels. On day 38, the first plasma samples collected after the 50% challenge SHIV dose escalation, revealed that five of the Group L animals had sudden breakthrough infection (large red dots in green boxes, **Figures 6**, **8**, animals L1, L5, L7, L9, and L10). **Figure 8** shows the anti-rgp41 (blue lines) and anti-P1 IgG responses (green lines) during the intravaginal SHIV challenges. There were no drops in the antigen-specific IgG responses just preceding the breakthrough infections; however, shortly after viremia became apparent, there was a marked boost in the vaccine-induced Ab responses, especially in animals L1, L11, and L12. Clearly, waning of vaccine-induced Ab responses did not account for the breakthrough infections seen after the SHIV dose escalation for challenge #8.

### Neutralizing and Binding Anti-SHIV Antibody Responses

**Figure 9:**
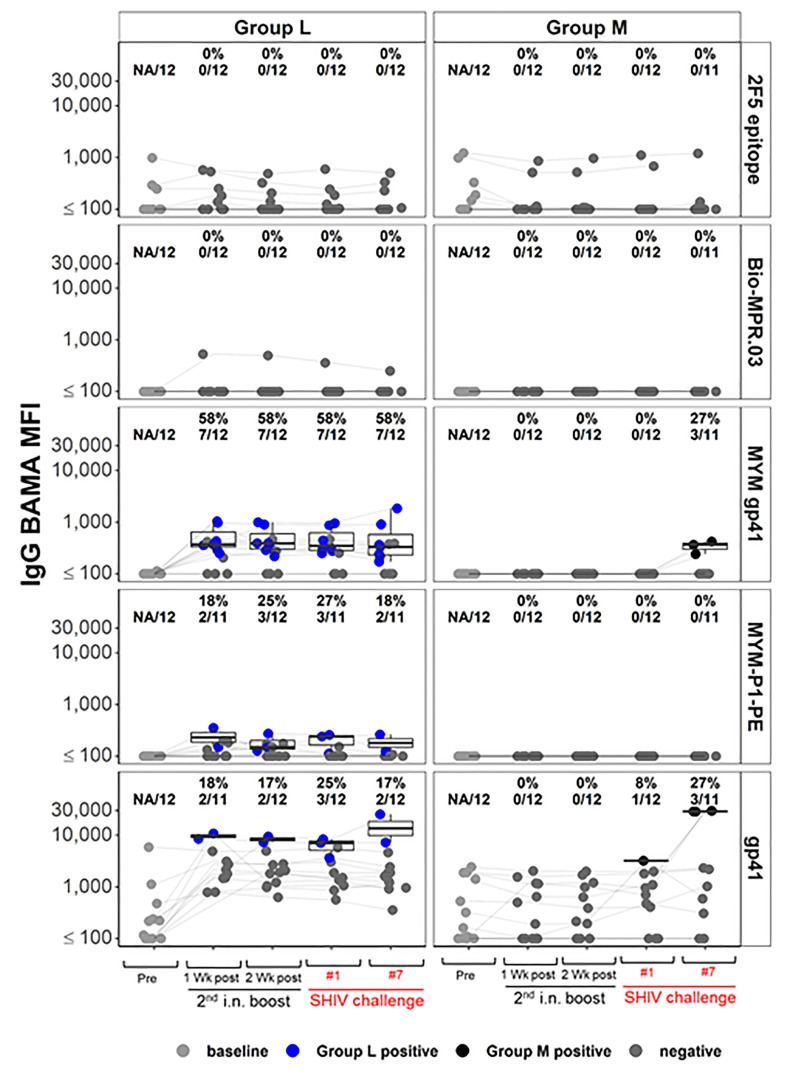
IgG Binding Antibody Multiplex Assay (BAMA) response magnitude measured by Mean Fluorescence Intensity (MFI), for the vaccine groups L and M, by time point and antigen (Methods). Dots are colored by response status with boxplots displaying distributions among positive responders. Baseline (pre) and negative samples are shown in shades of grey color; blue and black dots show positive samples for Groups L (immunized with the combination of virosome-P1 + virosome-rgp41) and M (given placebo virosomes), respectively. Response rates [percent positive, and ratio of number of positive samples/total number of samples] are shown for each combination. Lines join the same animal over time. High background reactivity against some gp41 antigens is notable for some animals.

Next, we assessed whether Group L vaccinees had developed nAbs against the challenge virus, the tier 2 SHIV_SF162P3_. None were detected at any time points in the plasma/serum samples examined (data not shown). This finding prompted us to examine the epitope specificity of the vaccine-induced IgGs by Binding Antibody Multiplex Assay (BAMA); the data are shown in **Figure 9**. Consistent with the lack of nAb responses in the functional assays, no reactivity was found against the 2F5 epitope (56) by IgG BAMA in Group L animals (**Figure 9**, top panels). This epitope, recognized by the broadly neutralizing mAb 2F5, is located in the MPER, and reactivity to this epitope would have been expected in animals vaccinated with virosomes displaying the P1 peptide. However, the experimental RMs had high background reactivity, including controls in Group M (**Figure 9**), possibly due to antigen mimicry between HIV gp41 regions and microbiome antigens (57, 58). For Group L, the anti-gp41 antibody responses were generally much higher during the vaccination phase compared to anti-P1 antibody responses (panels MYM gp41 versus MYM-P1-PE; **Figure 9**). In parallel, plasma IgA responses were also tested by the BAMA methods; no responses were found (data not shown).

IgG responses specific for the rgp41 antigen were tested in RMs of Groups L and M (**Figure 9**, 3^rd^ row of panels from top). No background reactivity was detected in any preimmune samples. Among Group L vaccinees, 7 out of 12 RMs mounted such IgG reactivity during the vaccination phase (blue symbols, **Figure 9**; 3^rd^ left panel). After the SHIV challenges resulted in infection, these specific anti-rgp41 IgG responses formed *de novo* in 3 out of the 11 control Group M animals.

### Fcγ-Related, Antigen-Specific Functional IgG Activities

**Figure 10:**
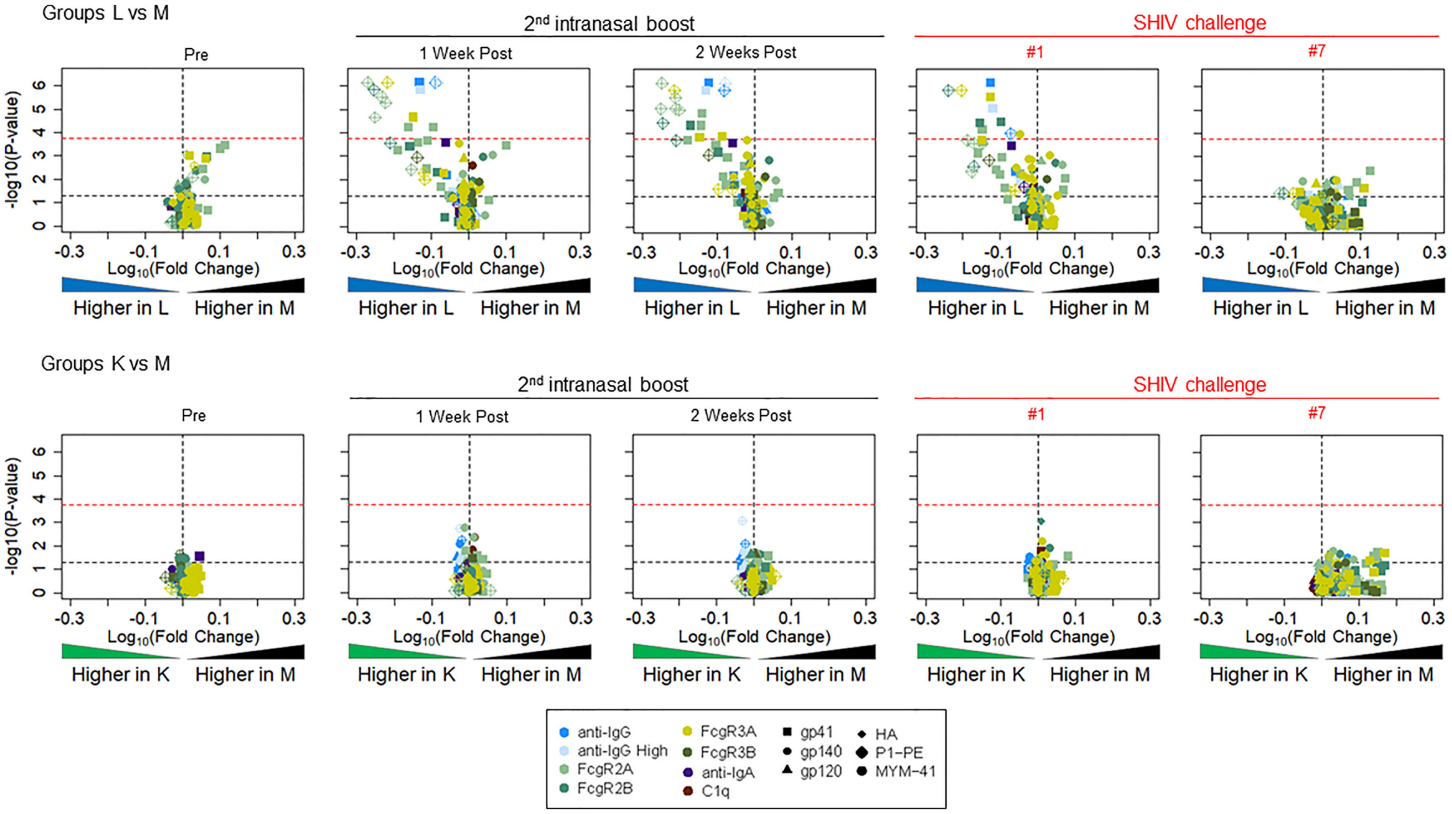
Volcano plots depicting fold change and significance of differences in Fc Array antibody features between immunization Groups L (top, given virosome-P1 + virosome-rgp41) and K (bottom given single-agent virosome-P1) and the control arm (Group M given placebo virosomes). Symbol shapes indicate Fc specificity and color indicates Fc characteristic. Horizontal lines indicate statistical significance by Mann-Whitney U test, with p = 0.05 indicted by the black dotted line, and a Bonferroni-adjusted significance threshold indicated in the red dotted line. Group comparisons of antibody profiles at time points including pre-immunization (pre), one or two weeks after the 2^nd^ intranasal boost, the day of the 1^st^ SHIV challenge, and the day of the 7^th^ SHIV challenge are reported; please see **Figure 3**.

Next, we performed Fc array analysis to examine the IgG features between Groups L vs M and K vs M. Briefly, plasma from the experimental animals at the time points indicated were incubated with beads conjugated to target antigens of interest followed by detection with FcγR, lectins, as well as IgG and IgA detection reagents. The data are presented as volcano plots that depict the fold change and significance of differences between the experimental groups versus control Group M (**Figure 10**). Between one week and two weeks after the second i.n. boost, Group L showed clear, specific reactivity compared to Group M; significant differences were also present on the day of the first SHIV challenge (**Figure 10**, top row, 4^th^ panel). The difference was no longer noticed on the day of SHIV challenge #7. In contrast, none of the time points showed any significant differences in volcano plots comparing Group K vs Group M.

**Figure 11:**
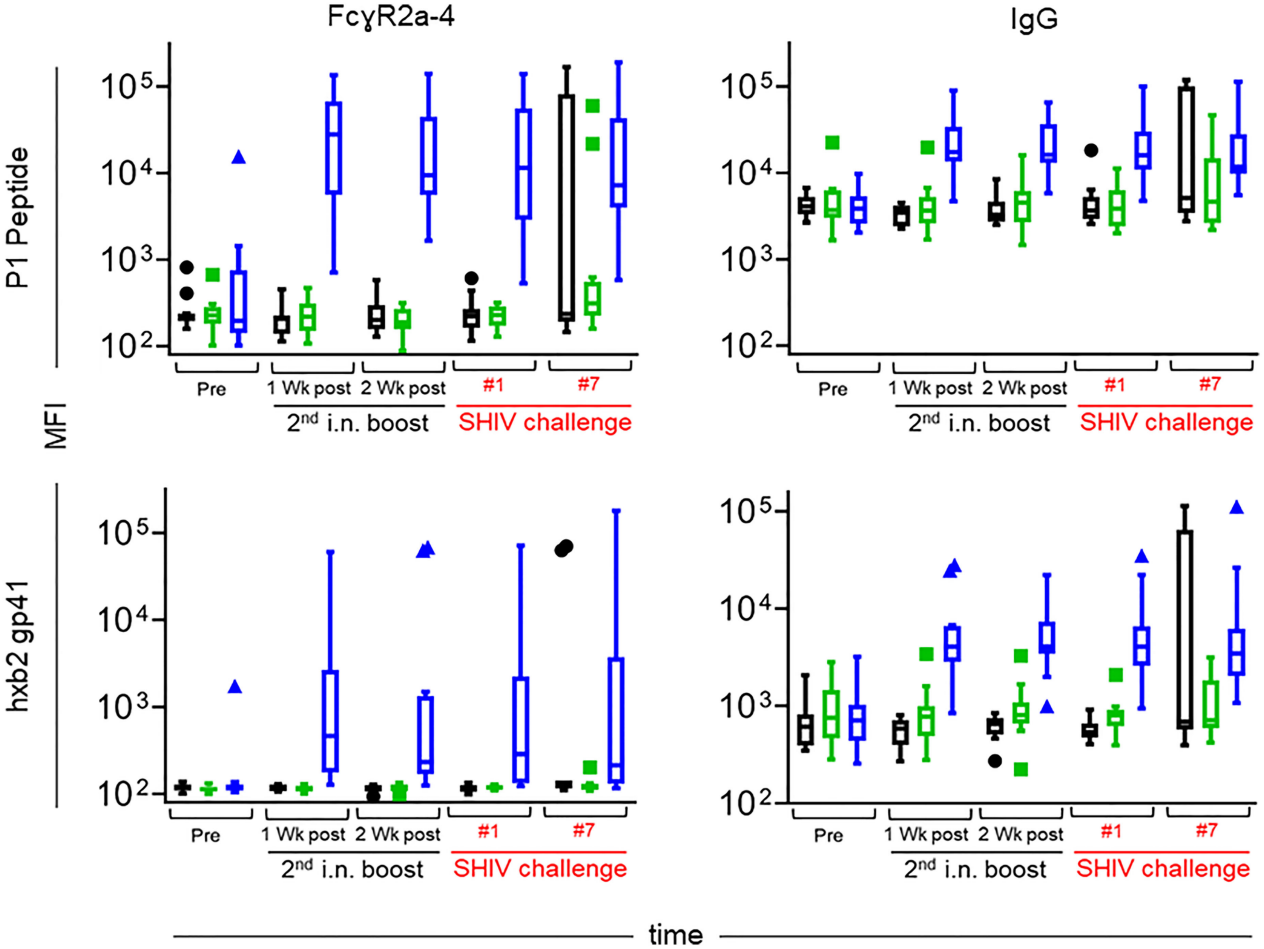
Levels of FcγR2a-4 binding (left) and total IgG (right) specific for the P1 peptide used as an immunogen (top) and recombinant gp41 from the HxBc2 strain (bottom) over time. Immunization groups are indicated in color; green, Group K; blue, Group L; black, Group M. Time points include pre-immunization (pre), one and two weeks after the 2^nd^ intranasal boost, the day of the 1^st^ SHIV challenge, and the day of the 7^th^ SHIV challenge (please see **Figure 3**). Median fluorescent intensities (MFI) are reported for each antibody characteristic; group medians are represented by the bar with Tukey’s box and whiskers.

We ascribe the differential outcome of the Fcγ arrays, and the functional assays for Fcγ-related activities, to differences in assay sensitivity, with the Fcγ array being significantly more sensitive. Overall, the volcano plots revealed significant differences between vaccinees in Group L and controls in Group M with regards to antigen-specific FcγR2A, FcγR2B, FcγR3A, and FcγR3B. **Figure 11** highlights specific reactivity to P1 peptide (top panels) and rgp41 (bottom panels). The three experimental groups are color coded as in other figures (Black, Group M; green, Group K; blue, Group L). After the second i.n. boost and on the day of challenge #1, the levels of antigen-specific IgG binding to FcγR2A-4 are increased in Group L vaccinees, compared to the levels in the other two groups.

Prompted by the finding of increased antigen-specific IgG to FcγR2A, we sought to correlate increased binding with functional activities. FcγR2A is preferentially expressed on phagocytes, including macrophages, monocytes, neutrophils, and dendritic cells, and plays a role in phagocytosis. Thus, we examined plasma samples for ADCP and ADNP.

**Figure 12:**
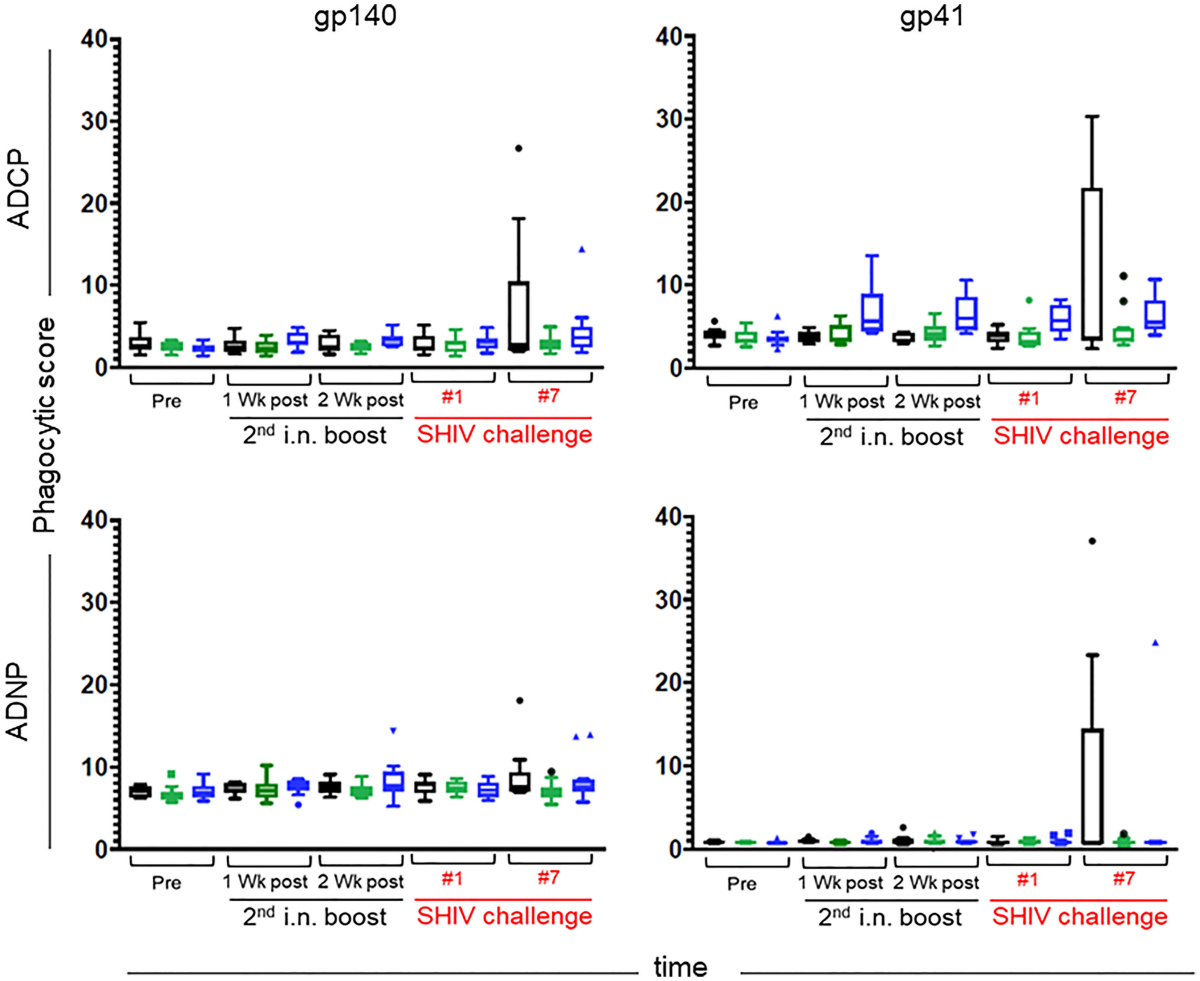
Antibody-dependent Cellular Phagocytosis (ADCP) (top) and Antibody-dependent Neutrophil Phagocytosis (ADNP) (bottom) were assessed against gp140 SHIV_SF162p3_ (left) and the recombinant Mymetics rgp41 (right) antigens. Immunization groups are indicated in color; green, Group K; blue, Group L; black, Group M. Time points include pre-immunization (pre), one and two weeks after the 2^nd^ intranasal boost, the day of the 1^st^ SHIV challenge, and the day of the 7^th^ SHIV challenge (please see **Figure 3**). Phagocytic scores are reported for each assay; group medians are represented by the bar with Tukey’s box and whiskers.

Overall, antigen-specific functional activities of the samples were low, with only a few data points above the negative control. On the day of challenge, neither ADCP nor ADNP were above background (**Figure 12****)**. Importantly, the samples with increased ADCP at week 33 (four weeks after the initiation of SHIV challenges) also demonstrated increased ADNP activity, suggesting the observed responses were vaccine-induced, as ADNP and ADCP activity often track together. Although Ab titers were not evaluated as part of the ADCP/ADNP assays, the weak functional responses coupled with the lack of detectable antigen-specific glycan suggests that relatively low-titer responses were elicited in the vaccine trial.

### Mucosal Antigen-Specific IgG + IgA Responses

**Figure 13:**
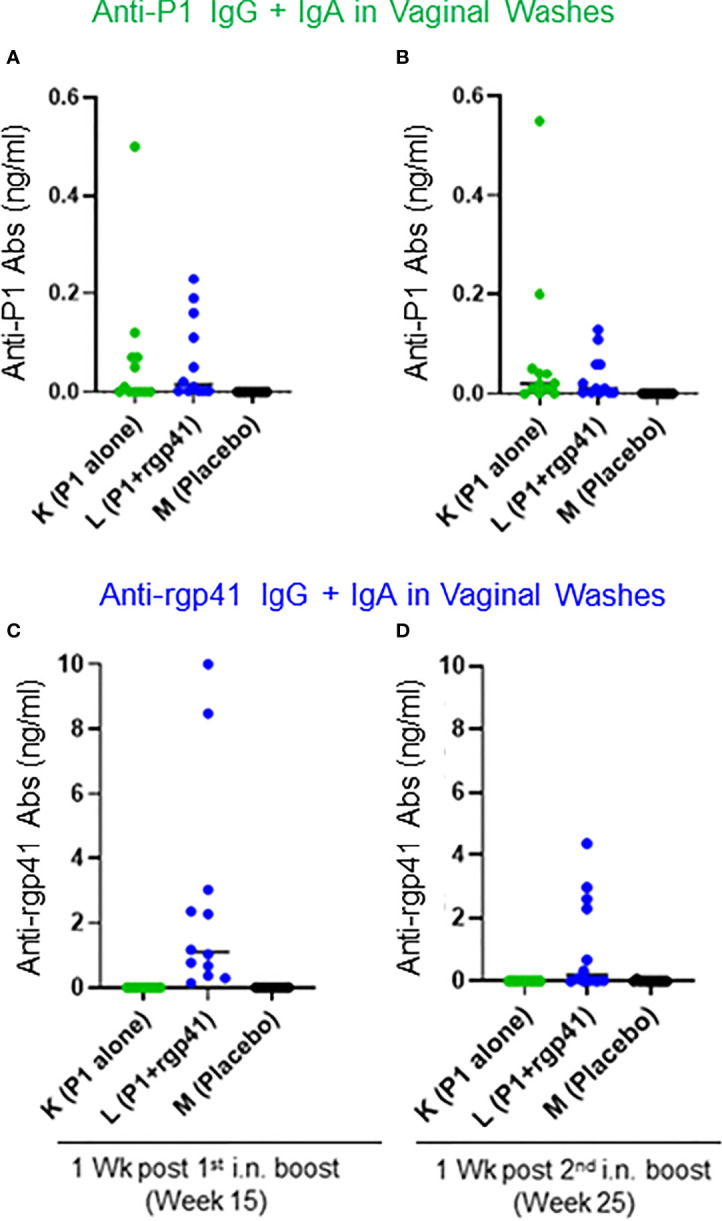
Antigen-specific antibody concentrations (ng/ml) measured in vaginal washes with the Imperacer^®^ assay (Methods). Group K (green circles) was immunized with single-agent virosome-P1 (P1 alone), Group L (blue circles) received the combination of virosome-P1 plus virosome-rgp41 (P1+rgp41), whereas Group M was given placebo virosomes (black symbols). **(A–D)** The sum of antigen-specific IgG plus IgA is given at the weeks after intranasal (i.n.) boosts indicated; **(A, B)** anti-P1 responses, and **(C, D)** anti-rgp41 responses.

Finally, we sought to determine the concentrations of antigen-specific IgG + IgA in vaginal washes using the ultrasensitive Imperacer^®^ assay (Methods) that takes advantage of PCR amplification in the last step of an ELISA. This assay allows measurements of Ab concentrations with a sub-ng/ml sensitivity. Vaginal washes were collected at weeks 15 and 25, which corresponds to one week after the first or second i.n. boosts, respectively. Only the sum of the IgG + IgA responses could be determined (**Figure 13**). Week 25 corresponds to four weeks prior to the first intravaginal SHIV challenge; no vaginal wash samples were collected after this time point to avoid introducing microabrasions that could compromise mucosal barrier integrity. Of note, absolute antigen-specific Ab responses were higher in Group L compared to Group K – possibly explaining the initial vaccine protection seen in Group L vaccinees but not in Group K animals.

## Discussion

Here we demonstrate that i) the combination of the HIV gp41 virosomes, i.e., virosomes-P1 plus virosomes-rgp41, achieved significant protection in Indian-origin RMs during the SHIV Challenge Phase I; ii) analysis of correlates of protection revealed no nAb responses, consistent with lack vaccine-induced anti-gp41 MPER Abs. However, by Fc array analysis, protection in Group L was significantly associated with increased FcγR2/3(A/B) across several time points compared to control Group M; iii) protection in Group L was lost in Challenge Phase II when the virus inoculum was increased by 50%; iv) estimates of the SHIV challenge inoculum in comparison with vRNA levels founds in the semen of men with acute HIV infection indicated protection against intravaginal challenge in the Indian RMs was at a high level as long as the SHIV inoculum did not exceed HIV RNA levels in semen of men with acute HIV infection by >100 fold (16); and v) single-agent virosomes-P1 provided no protection throughout Challenge Phases I and II and thus cannot be considered for clinical development. Importantly – during Challenge Phase I – we have confirmed that the combination of virosomes-P1 plus virosomes-rgp41 protects significantly against intravaginal SHIV challenges. Thus, the safety and efficacy of the gp41 virosomal platform, described earlier for Chinese-origin RMs, has been confirmed in Indian macaques.

Analysis of the correlates of protection in Group L using serum/plasma samples did not reveal significant Ab responses to the MPER epitope, a possible explanation for the lack of nAb responses. Furthermore, no significant ADCC (data not shown), ADCP or ADNP activities were detected in the vaccinees’ serum samples. However, Fc array analysis revealed a significant association between the protection in Group L during Challenge Phase I with increased FcγR2 or FcγR3/AB expression at several time points in comparison to control Group M. Cell-mediated immune responses were not measured and are unlikely to be significant following the i.m. priming/i.n. boosting vaccine strategy. In summary, our new finding is the positive correlation of Group L protection during Challenge Phase I with FcγR status.

Why did most of the Group L animals lose protection when exposed for the first time to a 50% higher SHIV challenge dose? It appears that the vaccine-induced Ab defenses became overwhelmed - akin to a dam suddenly inundated by a flash flood. Of note, eight RMs in Group L became viremic one week after being confronted with the higher challenge virus dose, while no new SHIV acquisitions occurred in the control Group M. This situation let us to postulate that the antigen load in the higher Challenge Phase II inoculum tilted the balance such that vaccine-induced Ab defenses could no longer hold the invading infectious virus at bay. In the current RM study, the gp41 content of the virus inoculum during the challenge Phase I was approximately 0.127 ng/ml and 0.193 ng/ml during Phase II, respectively (estimates from gp41 ELISA and the virus dilution factor). Based upon the vaginal wash samples for which the specific IgG + IgA content was determined (**Figure 13**), antigen-specific antibodies at the mucosal front line were at best 2-3 fold in excess over the gp41 load in the virus inoculum. Given these experimental conditions, a 50% increase in the antigen load in challenge inoculum was sufficient to disturb the delicate balance between antibodies and antigens, like a dam no longer high enough to stem the incoming viral antigen flood.

The protection in Group L, ranging from 78 to 87 percent depending on the readout parameter, begs the question whether the combination of gp41 virosomes used for this Group will likely be protective in preventing HIV transmission in humans. We compared the vRNA content of the SHIV inoculum with published reports of HIV RNA levels found in semen specimens of infected men at different stages after HIV acquisition. According to one estimate (17), during Challenge Phase I, the SHIV RNA content was 70,000 times higher than the median HIV RNA content in human semen. When the SHIV dose was increased for Challenge Phase II, the challenge dose given to the Indian RMs exceeded the HIV RNA content in infected men’s semen by approximately 105,000 fold. Another study by Pilcher et al. (16) estimated HIV RNA content in semen of infected men as function of time after becoming infected.

The current HIV virosomal vaccine MYM-V201 that has no added adjuvant can elicit a front-line mucosal defenses that could prevent male-to-female HIV transmission, provided the viral RNA levels in the men’s semen is low, as in those observed subjects with chronic HIV infection when HIV RNA copies may range between 100 to 10,000 copies/ml (16, 17, 59) representing ~50 to 5,000 virions/ml. The latter can be largely outnumbered thousands of times by the vaccine-induced anti-gp41 antibodies. However, preventing HIV transmission from acutely infected men with 100,000 to 2 million RNA copies/ml in their semen may be more difficult and require a vaccine formulation triggering stronger mucosal antibody defenses. Assuming that each virion has no more than 15 Env gp160 trimers on its surface (60) or 45 gp120/gp41 proteins, then 1 million virions/ml in the semen would represent ~45 million molecules of gp41/ml.

What are the major differences of the virosome vaccine efficacy studies performed in Chinese RMs versus Indian-origin macaques? The earlier study performed in China had yielded 100% protection of a small group of animals against repeated intravaginal SHIV challenges, using the same strain SHIV_SF162P3_. None of the animals given virosomes-P1 plus virosomes-rgp41 by two i.m. priming immunizations followed by two i.n. boosts seroconverted to SIV Gag, an antigen not present in the vaccine. Of note, sterilizing immunity was not achieved in all Chinese RMs, although viremia blips were low and then disappeared. During Challenge Phase I in the Indian RMs, a high degree of protection was also achieved, although not all animals remained below the threshold of 10,000 plasma vRNA copies/ml typically associated with seroconversion after the acute viremia phase. A key difference between the two studies is the age of the females. In China, the animals were 2.5 years old on average at the start of the vaccination, just at the age of becoming sexually mature. None had any offspring, and none had been mated. In contrast, the Indian-origin RMs available were older, with a mean age of 7.8 to 9.6 years depending on the experimental group and had diverse reproductive histories. This significant age difference could have influenced vaccine immunogenicity. It is becoming clear that older animals do not have the same levels of antiviral immune responses when compared to juvenile/young ones, resulting in better protection of young animals against virus challenges via the intrarectal (61) or intravaginal routes (Dr. Genoveffa Franchini, personal communication; Bissa et al., submitted). An additional variation between the studies performed in China versus Texas is environment, likely associated with differences in the microbiota, which are increasingly recognized as a determining factor for vaccine immunogenicity (62, 63); reviewed (64). Lastly, while all six control Chinese RMs became viremic (14), this was not the case in our more diverse, larger control Group M consisting of much older Indian-origin RMs. After the initial seven ivag SHIV inoculations (Challenge Phase I), followed by 15 SHIV inoculations at the 50% increased dose (Challenge Phase II), three Indian RMs in control Group M had remained aviremic. We decided to go beyond the original request to repeat the earlier study (14) and administered 1 ml of undiluted virus stock as a single, high-dose challenge to animals that were still aviremic at the end of both Challenge Phases I + II. Of the three aviremic Group M RMs, one animal (M8) became viremic and PSI positive; this result had no impact on the conclusion of the study’s Challenge Phases I & II.

Vaccine efficacy of Group K given single-agent virosomes-P1 needs to be discussed briefly. This group showed no statistically significant protection throughout the entire course of the study, including Challenge Phase I. As such, virosome-P1 alone is no longer considered a potential candidate for clinical development. Of note, the titers of anti-P1 Ab levels were low, and no anti-MPER Ab responses were detectable in the epitope mapping analysis. Such, the single-agent virosomes-P1 vaccine was neither immunogenic nor protective. The epitope analysis also revealed high background levels against P1; the latter may be due to the recently described cross reactivity between gp41 antigens and bacterial antigens (57, 58, 65). As such, the immunogenicity of virosome-P1 may have been compromised by the tolerogenic effects of cross-reactive bacterial antigens.

The low immunogenicity of the combined HIV-1 virosomal vaccine MYM-V201 (see **Figure 2**) in Indian RMs seen in the current study, particularly for the P1 peptide antigen, contrasts with the original studies in Chinese macaques (14) and the human Phase 1 trial in healthy women (15), for which good anti-P1 antibody responses were reported. In women, a single injection of the virosomal single-agent MYM-V101 containing only unadjuvanted virosomes-P1 led to > 90% seroconversion for the specific serum IgG and IgA, and serum titers and vaginal antibody levels were high with peaks after the third vaccination corresponding to the first i.n administration.

We cannot exclude the possibility that different methods and reagents could have influenced the outcome for the different studies. Another potential explanation may be related to genetic differences between Indian and Chinese macaques. In addition, the two NHP studies were conducted in different geographical locations (China versus USA), with different water and food supplies and animal housing conditions, all of which may have influenced the microbiota of mucosal tissues and skin. Cross-reactivity between antibodies recognizing host commensal microbial antigens and HIV gp41 has been described (65), and homologies between human host proteins and HIV gp41 antigen (57, 58) could contribute to reducing the antibody responses toward gp41 vaccinal antigens.

The vaccine efficacy study described here used MYM-V201 (virosomes-P1 plus virosomes-rpg41) without additional adjuvants. The virosomal platform by itself has self-adjuvanticity mostly due to the influenza virus-derived HA antigens. In addition, a new function for peptide P1 has recently been discovered: its ability to act as a mucosal adjuvant for unrelated antigens (66). Given the sudden loss of protection when the SHIV challenge dose was changed at the start of Challenge Phase II, adding additional viral antigens could be considered to increase the number of viral targets and thus overall immunogenicity. The unadjuvanted virosomal platform on its own is known to be a weak inducer of T-cell immune responses when compared to viral vectors, but the virosome formulation can be adapted to generate better cell-mediated immunity.

In sum, the combination of unadjuvanted HIV gp41 virosomes consisting virosomes-P1 plus virosomes-rgp41 was safe, immunogenic, and protective in Indian-origin RMs during Challenge Phase I. It should be noted that the challenge route was intravaginal – quite different from all other preclinical AIDS vaccine development studies that mostly used the intrarectal challenge route. We have assessed the amount of virus required to achieve persistent systemic infection in macaques using atraumatic challenges through different mucosal routes (25). The least amount of virus was required for the intrarectal route, followed by the intravaginal route, where eight times more virus was needed to achieve persistent systemic infection in naïve animals; the highest viral doses were needed for oral challenge in adult animals. Given that the vaccine-induced Ab responses appeared to be limiting as shown by the sudden collapse of protective defenses at very beginning of Challenge Phase II, it would be of interest to examine the vaccine efficacy of the gp41 virosomal platform against repeated low-dose intrarectal challenges in NHP models.

## Data Availability Statement

The original contributions presented in the study are included in the article/**Supplementary Material**. Further inquiries can be directed to the corresponding author.

## Ethics Statement

Approval for all procedures was received from the Institutional Animal and Care and Use Committee of the Texas Biomedical Research Institute.

## Author Contributions

RMR and SF designed the study. MA oversaw vaccine production and quality controls. SKL coordinated sample processing from animal studies. SKL, DH, and HKV performed *in vitro* assays. SKL conducted post-challenge virus titrations. MEA, VR, GA, GF, DCM, GDT, SS, and NLY performed assays and/or analyzed data for correlates of protection analyses. CG performed statistical analyses. DH, SKL, SF, and RMR wrote the report. All authors participated in the critical review of the data and manuscript.

## Funding

This work was supported by the Bill & Melinda Gates Foundation Grant OPP1111654 to RMR, NIH U19 AI142636 to RMR and SF, Collaboration for AIDS Vaccine Discovery, Vaccine Immune Monitoring Center (CAVIMC) grant OPP1146996, the National Institutes of Health/Office of Research Infrastructure Programs (NIH/ORIP) Grant P51 OD011133 to the Southwest National Primate Research Center (SNPRC) in San Antonio, TX, and National Institute of Allergy and Infectious Diseases (NIAID, Duke Center for AIDS Research (CFAR) grant P30 AI064518.

## Conflict of Interest

MA and SF are employees of Mymetics SA.

The remaining authors declare that the research was conducted in the absence of any commercial or financial relationships that could be construed as a potential conflict of interest.

## Publisher’s Note

All claims expressed in this article are solely those of the authors and do not necessarily represent those of their affiliated organizations, or those of the publisher, the editors and the reviewers. Any product that may be evaluated in this article, or claim that may be made by its manufacturer, is not guaranteed or endorsed by the publisher.
