# Supplementary material for: Cooperation Between Systemic and Mucosal Antibodies Induced by Virosomal Vaccines Targeting HIV-1 Env: Protection of Indian Rhesus Macaques Against Low-Dose Intravaginal SHIV Challenges
Source: Front Immunol. 2022 Feb 22;13:788619. doi: 10.3389/fimmu.2022.788619 (PMC8902080; doi:10.3389/fimmu.2022.788619)
Supplement: Supplementary file 1 [file DataSheet_1.docx]

**SUPPLEMENTARY INFORMATION**

| Supplementary Table 1 \| Antigens used in the Fc Array, organized by protein/peptide source. | | | |
| --- | --- | --- | --- |
| **Immune-Tech** | **Duke PPF** | **NIH AIDS Reagents** | **Mymetics** |
| gp41 HxBc2 | B.6240 gp140C/293F | gp140 BR29 | P1-PE synthetic lipopeptide |
| gp120 JRCSF | C.CH505TF gp140/293F |  | MYM-41-M0 truncated recombinant protein (rgp41) |
| gp120 MN | C3347_11 gp140C.avi |  |  |
| gp120 PVO | CNE5 gp140C.avi/293F |  |  |
| gp140 Du151 | QH0515.gp140C.avi |  |  |
| HIV-1 gp41 MN (*E.coli*) | 9004S gp140C.avi |  |  |
| gp120 SHIV_SF162P3_ |  |  |  |
| gp140 SHIV_SF162P3_ |  |  |  |
| HA1(A/Memphis/1/1987(H1N1)) |  |  |  |
| HIV-1 gp41 ectodomain |  |  |  |
| gp140 SF162 |  |  |  |

Duke PPF, Duke Protein Production Facility

| Supplementary Table 2 \| Reagents used to characterize the Fc domains of antibodies in the Fc Array. | | | | | | |
| --- | --- | --- | --- | --- | --- | --- |
| Antibodies/Complement | aRh IgG | aHu IgA* | C1q |  |  |  |
| Human FcγR | FcγRIIAH | FcgRIIAR | FcgRIIB | FcgRIIIAF | FcgRIIIAV | FcgRIIIB NA1 |
| Rhesus FcγR | rFcγR2A-4 | rFcgR2A-3 | rFcgR2A-2 | rFcgR2B-1 | rFcgR3A-1 |  |
| *Note that the anti-Hu IgA antibody (aHu IgA) from Southern Biotech was evaluated and found to be both cross-reactive with rhesus IgA and demonstrated improved signal as compared to specific anti-RhIgA detection antibodies tested. aRh IgG, anti-rhesus IgG. | | | | | | |
